# Searching for migration: estimating Japanese migration to Europe with Google Trends data

**DOI:** 10.1007/s11135-022-01560-0

**Published:** 2022-11-14

**Authors:** Bert Leysen, Pieter-Paul Verhaeghe

**Affiliations:** Department of Sociology, Vrije Universiteit Brussel, Pleinlaan 2, Room 2.05, 1050 Brussels, Belgium

**Keywords:** Big data, Migration, Japan, Forecasting

## Abstract

In recent research, Google Trends data has been identified as a potentially useful data source to complement or even replace otherwise traditional data for predicting migration flows. However, the research on this is in its infancy, and as of yet suffers from a distinctive Western bias both in the topics covered as in the applicability of the methods. To examine its wider utility, this paper evaluates the predictive potential of Google Trends data, which captures Google search frequencies, but applies it to the case of Japanese migration flows to Europe. By doing so, we focus on some of the specific challenging aspects of the Japanese language, such as its various writing systems, and of its migration flows, characterized by its relative stability and sometimes limit size. In addition, this research investigates to what extent Google Trends data can be used to empirically test theory in the form of the aspirations and (cap)ability approach. The results show that after careful consideration, this method has the potential to reach satisfactory predictions, but that there are many obstacles to overcome. As such, sufficient care and prior investigation are paramount when attempting this method for less straightforward cases, and additional studies need to address some of the key limitations more in detail to validate or annul some of the findings presented here.

**Supplementary Information:**

The online version contains supplementary material available at 10.1007/s11135-022-01560-0.

## Introduction

Migration studies remain hampered by several issues, key of which is the limited availability of reliable and up-to-date migration data (Willekens et al. 2017). Ahmad-Yar and Bircan (2021) identify several issues, from the multiplicity of measuring flows and defining stocks by governments and organizations, to the delay with which data is published. Furthermore, there is no comprehensive information on why people migrate. These compounding issues make migration predictions difficult and have an immediate impact on both policy and research.

During the past decades, however, new forms of data have emerged, primarily centered around the use of the internet and devices connected to it. Central are so-called *big data* defined as an “information asset characterized” by “High Volume, Velocity and Variety” (De Mauro et al. 2016). Important for international migration are sources where either “the primary usage is for geolocation” such as mobile device GPS signals or geotags on social media, or data with a location component as part of its “digital exhaust” (De Backer 2014), allowing people’s movements to be tracked.

There has been an increasing interest from migration scholars in using big data originating from social media (Dekker et al. 2016), such as LinkedIn (State et al. 2014), Twitter (Hsiao et al. 2020; Zagheni et al. 2014) or Facebook (Spyratos et al. 2019; Vespe et al. 2018; Zagheni et al. 2017), but also from mobile phones (Blumenstock 2012). *Google search data* too is fertile soil for research due to Google’s popularity as a search engine and its free-to-use analytics. Applications range from economics, tourism, medicine to health (Jun et al. 2018; Li et al. 2021). Also, migration research aims to improve models for predicting migration flows through Google search data with some success (Böhme et al. 2020; Wanner 2021).

Search data also hold promise for theoretical reasons. Scholars have started to approach migration as a combination of *aspirations* to migrate and the *ability* to do so as elements preceding any form of migration (de Haas 2021). Aspirations are brought about through their interaction with the migration environment and an individual’s characteristics (Carling and Schewel 2018). Yet gaining comparative insight into people’s migration intentions and aspirations remains challenging. Some research uses the Gallup World Poll (GWP) to measure country-level aspirations (Docquier et al. 2014; Laczko et al. 2017; Migali and Scipioni 2018). Yet, the GWP has problems as its few questions can be difficult to interpret (counterfactual) and only inquire about permanent migration (Carling and Schewel 2018). Also, accessing these micro-data is expensive, thus barring a wider audience with limited resources.

This is where free-to-access online search data may serve as an alternative to capture migration intentions and aspirations. International migration is a major decision for individuals or households. These decisions and subsequent preparations are accompanied by a search for information to facilitate this movement (Willekens et al. 2017). These searches thus reflect to some extent the *aspirations to migrate*, taking place *before* actual mobility.

More research is necessary to determine to what extent Google search data can be a valid source of data. Previous research has mainly focused on Google searches in Western languages and migration between Western countries (with Connor (2017) as a notable exception). So, despite the critical nature of both language and writing systems for this search activity how these aspects impact data and thus research on migration using this data, has been underexplored.

Therefore, the first aim of this study is to examine how language and the writing systems used by prospective migrants impact Google Trends data and its potential for estimating migration flows. We examine this question with the case study of Japanese immigration to Europe, more specifically to Germany, the United Kingdom, and France as three major European countries of destination, in addition to Belgium and the Netherlands as two smaller ones. Japan is linguistically homogenous, but its language is complicated, having two syllabaries and one logographic system, resulting in myriad ways of looking up information online.^1^ While non-Western languages are considered an additional complication in research utilizing Google Trends and are subsequently avoided (Böhme et al. 2020), we purposefully include its examination as a distinctive research aim. Moreover, Japanese migration to Europe as a topic in itself is understudied, both in English and Japanese language research. All these elements combined make Japanese immigration a compelling but challenging case to examine Google search data for predicting immigration flows.

The second aim is to examine how well immigration from Japan to Europe can be estimated using Google search data based on the methods of preceding migration research. Here, we rely on the migration process framework as explained by Carling (2002; 2014) and de Haas (2014; 2021). We hypothesize that migration aspirations translate into an active search for information. This search for information can partly be captured by online search activity, such as in Google. And if more people are aspiring and later planning to migrate, more people should be searching for information. So, all else equal, this increase (or decrease if aspirations temper) in searches may be reflected in Google search activity which can be interpreted by Google Trends data. Lastly, the increase or decrease in Google search activity may consequently reflect actual (subsequent) movement.

## Migration theories and alternative data sources

Migration as a multidimensional phenomenon has long eluded coherent theorization. Traditional theories on the initiation of migration focus one-dimensionally on economic factors (Massey et al. 1993). More recent research similarly focuses on specific drivers of migration, such as socioeconomic (e.g., education), institutional (e.g., migration policies and civil rights) and sociocultural factors (e.g., social networks, cultural ties). This multidimensionality reflects the inherent complexity of migration (Bijak 2011; Castles 2010; Czaika and Reinprecht 2020). As such, theories that aim to explain why people migrate face continued criticism. Massey et al. already noted how migration studies lack a commonly accepted theoretical framework (1994) and more recently Amelina and Horvarth (2017) argued how linking migration studies to general social theory is a key challenge for the future of the field.

An attempt to address these critiques is the aspirations-(cap)ability approach, most notably proposed by Carling (2002) and elaborated upon by de Haas (2014; 2021). The framework goes beyond the one-dimensional focus on migration determinants by conceptualizing migration as a combination of aspirations to migrate and the ability to do so. Patterns of aspirations develop in the interaction of the migration environment with individual characteristics (Carling and Schewel 2018). Whereas Carling (2002) developed the framework to deal with ‘involuntary immobility’ (i.e., aspiring but unable to migrate), de Haas (2003; 2014; 2021) reframed ability as ‘capabilities’, based on Sen’s capabilities approach. He considers aspirations as a function of “people’s general life aspirations and perceived spatial opportunity structures” and capabilities as dependent on “positive and negative liberties” people experience (de Haas 2014, 2021). Migration aspirations are typically seen as static factors: one either aspires to migrate or one does not. And those with the ability to do so, end up migrating.

Yet migration aspirations themselves can be influenced by capabilities, which is why a more nuanced understanding of these dynamic aspirations is paramount. Some researchers have hinted at this dynamic nature. Migali et al. (2018) showed with GWP data how more people aspire (intend) to migrate than end up preparing for it in the next 12 months, illustrating that not everyone that wishes to migrate ends up moving. Carling and Schewel (2018) noted similarly how with increased specificity of migration-related questions in the GWP, the answers can differ greatly. While these studies recognize nuances in migration aspirations and preparations, empirical application remains limited largely due to the difficulty in capturing these distinctions.

A reason for these limited applications is the paucity of reliable and accurate data. Despite efforts by governments and organizations, traditional migration statistics have not improved notably (Ahmad-Yar and Bircan 2021). In an attempt to find alternatives to inadequate official data sources, researchers have turned to various forms of big data. Zagheni and Weber (2012) used Yahoo! e-mail data to estimate the rates of international migration. They discovered e-mail data have the potential to complement existing data for increased accuracy in developed countries. Together with Zagheni et al. (2017), these authors later measured migration stocks with Facebook advertising data containing socio-demographic data of its users. The authors see the potential of digital data, not only for migration but for investigating all kinds of demographic elements—finding it particularly promising for countries lacking the official infrastructure to track migration in an organized way. Zagheni et al. (2014) used Twitter data from a subset of about 500,000 users in OECD countries to infer migration patterns. They found that, although difficult to predict overall variability, Twitter data was useful for predicting significant turning points in migration trends. Around the same time, State et al. (2014) used LinkedIn data (geolocated career histories) to examine trends in the migration of professional workers. While the authors did not focus on prediction, the data showed levels of granularity that are difficult to find in national statistics, especially since these are typically not easy to compare cross-nationally. Combinations of these new data and traditional sources have also added depth to investigations. For instance, Yildiz et al. (2019) combine bilateral migrant stocks with Facebook monthly and daily active user data to construct a Bayesian hierarchical model for EU migration stocks. Other research uses the same social media data, adjusted for bias, and combines this with traditional survey data to produce so-called “nowcasts” of migrant stocks in the United States (Alexander et al. 2022).

Prediction with big data entered a new chapter with the help of data generated by internet search engines. In particular, Google, both due to its increasing popularity and it being the default search engine on lower-end smartphones,^2^ has been used frequently in academic research. Specifically, Google Trends, a platform that maps the relative popularity of search terms across different locations has been a crucial new data source. A pioneering application was by the hand of Ginsberg et al. (2009) who matched Google searches on the flu to actual levels of influenza. Since then, applications using Google Trends data to forecast events have proliferated in the fields of economics, tourism, medicine and health, to information technology (Jun et al. 2018; Li et al. 2021). More recently new fields of investigation have opened up, dealing with novel topics such as forecasting unemployment insurance claims following hurricanes (Aaronson et al. 2022), vaccine hesitancy and anti-vaccination sentiments in the context of Covid-19 (Pullan and Dey 2021), and cross-national investigations in more established areas, such as disease modeling of Covid-19 for a range of European countries (Sulyok et al. 2021).

Applications to migration studies followed suit. In 2016, a study by Vicéns-Feliberty and Ricketts analyzed searches of Puerto Ricans on migration to the United States, and to five states popular among Puerto Rican migrants (Vicens-Feliberty and Ricketts 2016). Based on Google search data, they found that different states were popular for different reasons (job-related reasons, family considerations, and political party). In 2017, Connor successfully tracked the movements of refugees by examining internet searches in Arabic for the word ‘Greece’ within Turkey, an important migration corridor into Europe (Connor 2017). Even trends within a single day could be discovered with hourly data. Kostakos et al. (2018), also focusing on refugees, investigated whether search data could improve the forecasting of their arrivals in Greece.

Böhme et al. (2020) showed how Google search information (in English, French, and Spanish) can successfully be used to predict bilateral migration flows as search hits seem to reflect the intention to migrate. The predictions with these data outperformed models based solely on traditional data, such as GDP and unemployment rates. The Google Trends Index the authors constructed was also used by Golenvaux et al. (2020) in the same languages to test a long short-term memory (LSTM) approach which included Google search data against a linear gravity model (a more traditional approach), and an artificial neural network (ANN) model. Both models were outperformed by the LSTM approach combining Google data, illustrating its potential. Wanner (2021) opted for a simplified approach to the above studies. Instead of using a long list of possible keywords and coopting these in more elaborate models, he used one key phrase in the dominant language of the country of origin (‘working in Swiss’) to predict the predominant labor migration from Spain, Italy, France, and Germany to Switzerland. By linear regression and taking into account specific periods of lag (i.e., a delay between when a search action is executed and when one actually moves), he successfully predicted to some extent migration flows from Spain and Italy albeit with less convincing results for France. Avramescu and Wiśniowski (2021) followed a similar approach with Google Trends Indexes in English and Romanian, constructing composite variables capturing the interest of Romanians migrating to the United Kingdom. Their indices for employment and education managed to match the trends of official migration statistics, proving the data’s potential for further research. Research by Fantazzini et al. (2021) on internal migration in Russia using Google data was less successful, although they did succeed in reducing forecasting errors by including the data in a larger model.^3^

These studies have advanced the examination of alternative data sources substantially. However, deeper empirical investigations of how Google search data may be integrated into furthering theory, such as the aspirations-(cap)ability framework (Carling 2002, 2014; Carling and Schewel 2018; de Haas 2021), are limited. In addition, barring a few exceptions, the research has suffered from a predominantly Western bias. Consequently, our understanding of the wider applicability covering other regions is still lacking.

## Japanese migration to Europe

Research into modern Japanese migration to Europe is primarily historical in nature, often dividing the narrative in pre- and post-World War II. Between the Meiji Restoration in 1868 and World War II, Japanese migration consisted first of a considerable flow of labor migration to the Americas. This movement can be explained by both the labor surplus in Japan following the economic turbulences during that time and the labor shortages on large sugar plantations such as in Peru, Hawaii, or Cuba (Adachi 2006; Moore 2010). A second flow was comprised of colonial migration to countries in East and Southeast Asia (Caprio and Jia 2009; Tamanoi 2006). Neighboring areas in Asia, increasingly subjected to Japan’s imperial rule, were typically seen as being home to ‘peoples to civilize’ (Prasenjit 2001; Robertson 2006) and as part of an industrializing framework of economic opportunities.

Compared to the flows to Asia and America, migration flows to overseas communities in Europe were much smaller. During that time, Western Europe was predominantly a destination for political leaders, intellectuals, or artists. It served as an area for cultural and artistic inspiration and for studying the economic and political blueprints for establishing a modern Japan. Based on figures from the *Kodansha Encyclopedia of Japan*, James Stanlaw (2006) estimated that the number of Japanese emigrants to Europe in the pre-World War II period (1868–1941) did not exceed 7,980. In comparison, the Korean Peninsula alone witnessed 712,583 Japanese arriving in the same period.

After the war, Japanese migration became predominantly economic in nature. Taking the center stage in this era are Japanese multinationals, typically featuring local headquarters or branch offices in Europe employing Japanese on a rotation base either as trainees or managers (Sedgwick 2001). A clear example is Toyota’s European headquarters in Belgium (founded in 1963 in Denmark) with various vehicle and engine manufacturing plants in addition to design and R&D centers across Europe (Toyota Motor Europe 2021). Following Bonacich’s theory (1972), Cheng and Katz (1998) consider these Japanese expats as “middleman minorities” living “close by each other, establish(ing) Japanese schools for their children, giv(ing) rise to neighborhood markets that speak Japanese and stock(ing) Japanese food, and in general maintain(ing) a distinctively Japanese community” (p.60).

Some of these subjects are reflected in the literature centered on Japan and migration. Post-war *Japanese internal (regional) migration* has been a topic of academic concern since at least the 1970s (Kornhauser 1976; Okazaki 1977; Oshiro 1976). *Migration policies* are covered as well. Recent examples include Sakanaka’s recent historical treatise (2021), Oishi, who analyzed Japan’s policies focusing on highly skilled migration (2012, 2021), or Hollifield and Orlando Sharp’s examination of Japan’s policy changes in its transition from immigration to emigration state (2017).

*Migration to Japan* has been researched predominantly in the context of post-colonial linkages and pre-War connections due to labor migration movements from Japan. Bartram (2000) brings labor migration to Japan into focus as a “negative case”. He makes an argument for the inclusion of the analysis of countries with low numbers of foreign workers in migration studies Morgan and colleagues (2016) analyze international marriages in Japan. By way of interviewing foreign spouses, the authors zoom in on the motivations behind these movements.

Closely related are *diaspora studies* such as work by Chitose (2022), Green (2010), and Nishida (2018) on the Brazilian diaspora in Japan. The Korean diaspora, often captured by the term *zainichi*, is examined by scholars such as Chapman (2009), Kim (2005), Lie (2008), Ryang (1997) in English, and Morita (1996), Mun (2007), Fukuoka (1993), Kang (2008), or Kang and Oguma (2008) among others in Japanese. Literature on the Japanese diaspora in Europe is less prolific, with notable exceptions, such as the pioneering work by Glebe (1986) on the Japanese community in Düsseldorf, Germany.

The inclusion of *Europe* in research dealing with Japanese migration chiefly centers on contrasting case studies, where a European country (often the United Kingdom; see for instance Phillimore et al. 2021; Sigona et al. 2021; Wakisaka and Cardwell 2021) is juxtaposed with Japan and topics such as migration law and policy, migration infrastructure, marriage migration, and interregional migration are analyzed in a comparative framework (Dzienis 2019; Estévez-Abe and Caponio 2022; Ishikawa 1999). However, research dealing with migration from Japan to Europe, especially quantitative research, is lacking to the best of our knowledge.

The research presented here aims to contribute to the three areas of study presented above. First, it aims to further the examination of the framework established by Carling (2002) and de Haas (2014; 2021) dealing with migration aspirations specifically, by making use of alternative data (Google Trends). Next, we widen the investigation of Google Trends by analyzing its applicability to other areas and languages (Japanese). Last, this research contributes to the topical lacuna of quantitative analyses of Japanese immigration to Europe.

## Data and methods

### Data

This study makes use of several datasets. A first dataset consists of official immigration figures. The first target was to obtain the monthly data for each country to construct a detailed analysis and examine the results based on different lags (in months) between search and movement. However, these data are not always readily available. The statistical agencies of the different countries were contacted by email with the request for access, but only the representatives of Belgium replied positively to this inquiry.^4^ The other countries stated that monthly numbers are not available and referred to the yearly data.^5^ Other research has implicitly encountered the same limitation and has successfully used yearly data instead (Böhme et al. 2020; Golenvaux et al. 2020). For yearly figures, OECD data proved to be more complete than Eurostat data when consulted. For instance, entries for Germany after 2008 were missing. When necessary, the data were supplemented with numbers of the national statistical agencies.^6^ The immigration flow data for the United Kingdom are based on the yearly “International Passenger Surveys”. It should be noted here that these survey numbers are not accurate immigration figures and are rounded to one hundred.

An additional analysis specifically considers university education mobility. For these flows, the yearly reports (“*Results of Survey on Status of Japanese Students Studying Abroad Based on Agreements and other Sources*” or 協定等に基づく日本人学生留学状況調査結果) by the *Japan Student Services Organization* (JASSO) are used. Only the U.K. is listed in each report as a destination, so the focus of analysis will be on this specific location.

Next, Google Trends data are used (trends.google.com). This tool allows extraction of the relative search frequency of one or a set of keywords input in google in a specific geographic entity. Data is available for free from 2004 onwards. The relative frequency is indicated by a number between 0 and 100 (low to high search intensity) and is provided for each month in the time series specified. Absolute frequencies are not made public due to privacy concerns. While Google’s market share in Japan is not as high as in the U.S. or Europe, it is still over 70% for the period January 2009 to December 2021 according to StatCounter^7^ and had been fairly consistent over the years.^8^ Statista puts the current market share at around 76%.^9^ Also, according to the International Telecommunication Union, the internet is widely accessible in Japan (2021).^10^ Access but more importantly market share remains a potential limitation. Low market share would not only impact the generation of usable data points in Google Trends, but more specifically make the data less representative of the overall population potentially biasing results.

Google Trends data are investigated for two periods: 2006–2019 and 2011–2019. Similar to previous research, 2006 is selected as a starting point because it coincides with a more widespread adoption of Google. We end with 2019 because, at the time of research, national statistics for 2020 were not yet available. We opt for this double approach because Google implemented an algorithm change in 2011. Depending on the keyword input, stark differences can be seen in the time series pre-and post-2011 data. Since Google Trends is a relative index, it is not possible to use the same dataset and investigate the post-2011 numbers separately as the numbers are in relation to all the data in the set. Each new period under investigation necessitates a fresh generation and extraction of Google search frequencies.

For the sections where only country names were used (see “Methods”), the data for Belgium is only considered until May 2018. Due to the popularity of the World Cup football game Japan-Belgium on July 2, 2018, any search action that only takes into account the Japanese word for ‘Belgium’ culminated in an excessive peak around this date, thus skewing all the related data.

Next, we make use of an existing keyword list generated by Böhme et al. (2020). This list has been successfully used in other research too (Golenvaux et al. 2020). In this study, the list is modified and translated to suit the specific context of Japanese immigration. Here, the focus is solely on the Japanese language. Despite mandatory English classes in the Japanese education system, English is not routinely used by native Japanese to the extent that it could realistically be captured by online search activities. As a consequence, Google searches in English would primarily capture the search activities of foreign nationals in Japan. Since these people are typically not included in official immigration statistics counting Japanese citizens entering a country, including non-Japanese Google searches in the Google Trends data would add additional bias to the analysis. As such, the focus is on Japanese language specifically to target the searches of Japanese nationals.

In addition, the Japanese language is sufficiently complicated to warrant a standalone investigation as it has several writing systems. (1) *Kanji* originates from Chinese characters and is mainly used for *kango* or Sino-Japanese words. Most nouns and parts of adjectives are written in *kanji* (e.g., ‘music’ 音楽, or the first character of ‘beautiful’ 美しい). (2) *Hiragana* is primarily used for grammatical suffixes of words (for instance endings to denote the past tense of adjectives or adverbs such as the aforementioned ‘beautiful’ 美しい ・美しかった・美しく) and grammatical elements in sentences (e.g. は can mark the topic of a sentence or indicate contrast). (3) *Katakana* on the other hand is mostly used for loanwords, scientific words, and other imported terminology such as IT-related jargon. While (4) *rōmaji* is rarely used by itself, it can be used to input Japanese on digital devices. Several systems of transcribing a Japanese pronunciation to Latin script exist. We only consider the Hepburn and *Nihon Shiki* systems here. The former is used primarily by non-native speakers, and the latter is the main system used by native Japanese speakers.

The specific difficulty with applying the Japanese language to research with Google Trends is twofold: first, the different writing systems are not always mutually exclusive. For instance, the same Japanese word for ‘beautiful’ can be written both in *kanji* and *hiragana* (きれいな or 綺麗な). Both versions of this word are commonly used although for most *kanji* is preferred due to the second complication: Japanese is rife with homophones (see supplementary data for examples).^11^ As such, using *kanji* would be the logical option for searching online, but being a logographic system as opposed to a simple alphabet, not all characters are equally well known. Their sheer number can make *kanji* difficult, so even well-educated Japanese typically have not memorized all of them.^12^ In case of ambiguity or uncertainty, one may opt to use *hiragana* when searching the internet.

### Methods

The method of clarifying this as it relates to our research is straightforward. Based on the keyword list by Böhme et al. (2020), 20 migration-related keywords were selected. This list is supplemented with ten keywords that focus on the specific Japanese migration experience, so centering around overseas study (e.g., ‘study’ or ‘scholarship’), expats (e.g., ‘insurance,’ ‘work,’ or ‘tax’), and overseas Japanese communities (e.g., ‘Japanese food’ or ‘Japanese Association’).

**Fig. 1:**
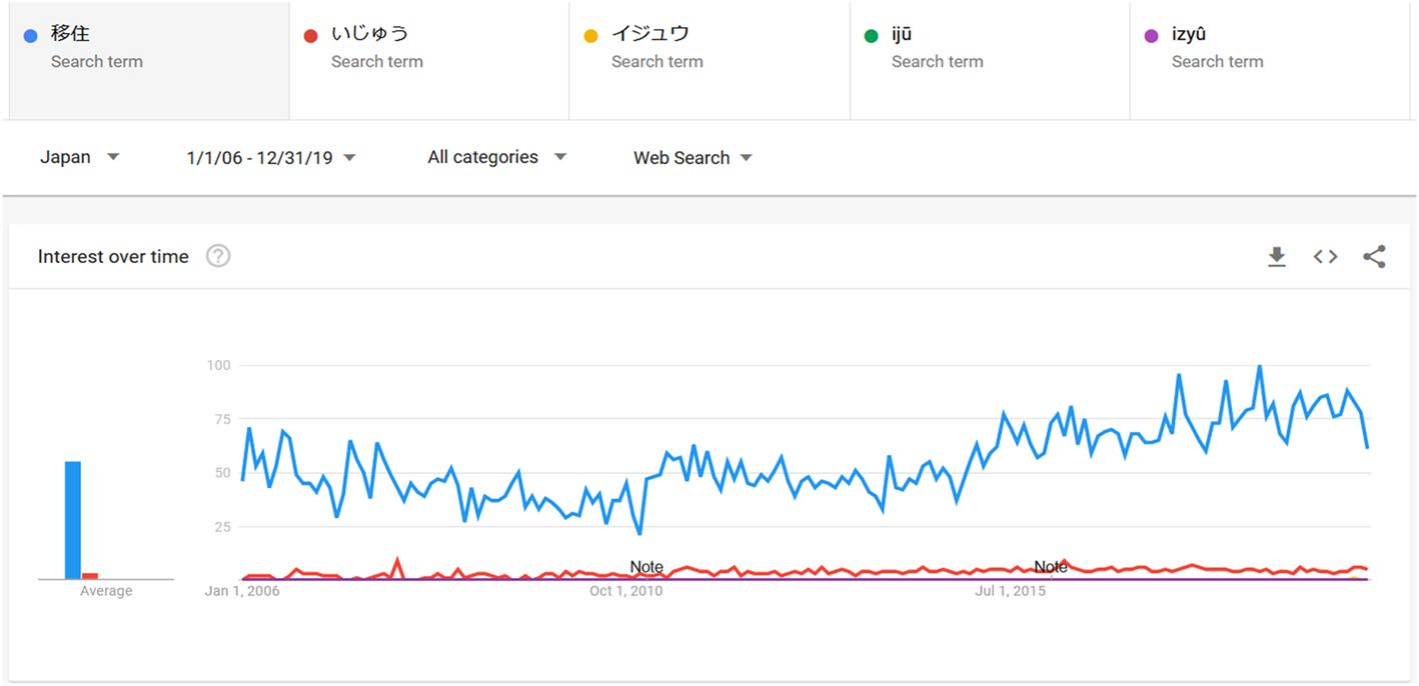
Google Trends image capture comparing the search frequencies of the same word in kanji, hiragana, katakana, rōmaji (Hepburn), and rōmaji (Nihon Shiki). Source: Image captured from trends.google.com

Each keyword is inputted and compared in Google Trends in as many ways as possible. Concretely this means that, when possible, the same word was input in (1) *kanji*, (2) *hiragana*, (3) *katakana*, (4) *rōmaji* (Hepburn system), (5) *rōmaji* (*Nihon Shiki* system) (see Fig. 1 for an example). Loanwords in *katakana* do not have a *kanji-*equivalent so this option is left out for these words, resulting in two sets of words: a) *kango* or Sino-Japanese words which have a *kanji* equivalent (24 words), and b) loanwords that are predominantly *katakana* and do not have a directly corresponding *kanji* (six words).

Next, the time series of the different inputs for every keyword in Google Trends are compared to come to an understanding of how these different systems impact the data that can be extracted.

To predict migration with Google search data, we start with the same keyword list by Böhme et al. (2020). Whereas research by Golenvaux et al. (2020) successfully used the list unmodified to predict immigration, to use it for Japanese migration it (a) needs to be adjusted to reflect the specific nature of Japanese migration and (b) needs to be translated taking into account the specificity of the Japanese language. Concretely, most words dealing with topics such as ‘asylum’ or ‘smuggling’ were deleted as these are not relevant to Japanese immigration to Europe, and words such as ‘insurance’ or ‘studying overseas’ were added. Also, words such as ‘migration’ and ‘migrating’, while different in English, are differentiated in Japanese only by grammatical sentence constructions (e.g., *ijū* and *ijū suru*). The words containing the meaning of the words do not include these grammatical differentiators. This means that these keywords are identical in Japanese.

Finally, following the findings of examining the different writing systems, the words are translated and transcribed resulting in a list of 90 words (Table 1). For some words, compound search terms are also constructed, both to boost measurable search frequencies by Google where results were lacking and subsequently to promote data extraction, and to address the issue of synonyms. For instance, we combined the words ‘consulate’ and ‘embassy’, and operated the search term as follows in combination with ‘Paris’: パリ 領事館 + パリ 大使館 (‘Paris consulate + Paris embassy’).

**Table 1:** List of 90 keywords

Japanese search term	Translation	Japanese search term	Translation
アパート + マンション	Apartment + apartment	年金	Annuity
インターンシップ	Internship	引っ越し	Moving (living places)
インフレ	Inflation	応募者	Applicant
お金	Money	応募者 + 募集 + 採用	Applicant + recruitment + employment
シェンゲン	Schengen	所得税	Income tax
チケット	Ticket (airplane)	採用	Employing
パイロール + 給与支払	Payroll	日本人	Japanese citizen
パスポート + 旅券	Passport + passport	日本人会	Japanese (expat) Organization
ビザ	Visa	日本人会 + 海外駐在員	Japanese (expat) Organization + expats
ビザ + 査証	Visa + visa	時給	Hourly wage
ビザ免除	Visa waiver	未登録	Unregistered
ホテル	Hotel	条件	Condition
不況	Recession	査証申請	Visa application
亡命	Flee	業務	Business
仕事	Work	求人	Recruiting
保険	Insurance	求人 + 欠員 + 空席	Recruiting + vacancies + vacancies
入国	Entry (in a country)	無職 + 失業	Unemployed + unemployed
最小限 + 最低限 + ミニマム	Minimal + minimum + minimum (wage)	留学	Study abroad
出入国管理 + 出入国	Immigration control + immigration	福祉 + 厚生	(social) welfare + public welfare
出国税	Departure tax	移住	(im)migration
切符 + チケット	Ticket + ticket	移動	(im)migration
到着	Arrival	移民	(im)migration
勉強	Study	移民 + 移住者 + 移住民 + 移住	(im)migration + (im)migrant + (im)migrant + (im)migration
募集	Recruiting	税務署	Tax office
医療保険	Medical/health Insurance	税理士	Tax accountant
収入 + 金儲け + 所得	Income + income + Income	税金	Tax
受益	Profit/benefit	税関	Customs
合法化	Legalization	組合	Association
和食	Japanese food	経済	Economy
国内総生産 + GDP	GDP + GDP	給料 + 手当	Salary + allowance
国外駐在 + 駐在	Expat + expat	脱税	Tax evasion
国籍	Citizenship	航空券	Airline ticket
国籍 + 国民性	Citizenship + nationality	補償	Compensation
国籍喪失	Loss of citizenship	要求	Request
在留邦人	Japanese overseas residents	観光客 + 旅客	Tourists + passengers
報酬 + 謝礼	Rewards + rewards	費用	(living) expenses
外国人	Foreigner	追放 + 送還 + 強制移住	Expulsion + repatriation + forced migration
大使館	Embassy	違法	Illegal
契約	Contract	雇い主	Employer
奨学金	Scholarship	雇用	Employment/job
定員	Capacity/quota (for applicants etc.)	雇用 + 職 + 就業 + 仕事	Employment/job + employment/job + employment/job + employment/job
密輸船 + 売人 + 運び屋	Smuggler + seller + carier	難民	Refugee
差別	Discrimination	非公認	Unofficial
帰休 + レイオフ	Lay off + retirement	領事館	Consulate
帰化	Naturalization	領事館 + 大使館	Consulate + embassy

For determining the statistical association of Google Trends, several approaches are examined. As a first step, a straightforward approach is used, following Wanner (2021). The keywords are inputted in Google Trends together with the Japanese word for each country. For instance ‘study (in) France’ would be translated into 留学 フランス. Monthly time series of Google Trends (ranging from 0 to 100) are downloaded for 2006 to 2019 and 2011 through 2019 and are aggregated for each year *t* in Japan (*ja*) as location.^13^ The resulting time series are labeled as bilateral Google Trends indexes (*GTIbil*_*jat*_). We estimate linear regression models via ordinary least squares method (OLS) to examine the relationship between immigration (*y*_*t*_ = the number of moves in year *t*), and the relative number of searches in year *t* conducted in Japan (*ja*), expressed by *GTIbil*_*jat*_.

The above analysis is repeated for specific keywords relating to educational mobility (e.g. ‘scholarship’ or ‘studying abroad’) in combination with the U.K. as country of destination. The general immigration numbers were replaced by those specifically for students based on JASSO’s collected data.

In a second step, we follow Golenvaux et al. (2020) and Böhme et al. (2020) and construct an interaction term consisting of additional Google Trends indexes: *GTIuni*_*jat*_ × *GTIdest*_*jat*_.^14^ Whereas the aforementioned authors construct one Google Trends index which aggregates the frequencies of all the keywords, we maintain the frequencies per keyword to examine the possible nuances between words. Although the assumption is that all associations of the words should follow the same direction, this needs to be confirmed by considering each word individually. *GTIuni*_*jat*_ is an independent variable containing the Google Trends values of the keywords by themselves for Japan during year *t* (i.e., not specifying the European destination). *GTIdest*_*jat*_ is the relative search intensity in Japan for the country names (e.g., ‘France’ but without another keyword). OLS linear regression is used for the periods 2006–2019 and 2011–2019 but with two predictors: *GTIbil*_*jat*_ + *GTIuni*_*jat*_ × *GTIdest*_*jat*_.

Compared to moving from Germany to France for instance, migrating from Japan to Europe requires more planning both due to the distance (both Euclidean and cultural) involved and the additional paperwork compared to within-Schengen movement. To capture this preparation phase, the models are run again with a one-year time lag (*y*_*t-1*_) for Germany, France, the Netherlands, and the United Kingdom. Because monthly data are available for Belgium, the number of lags is increased and delays of three, 6, 9, and 12 months between searching and moving are examined for this country.

In a third search action, we only focus on the country and city names. Instead of examining general searches, a built-in tool by Google Trends is used that categorizes searches in specific categories. The data are extracted based on four categories: (1) *all categories*, (2) *business and industrial*, (3) *jobs and education*, and (4)* law and government*. The resulting time series only take into account searches related to the specified categories and are thus not limited to exact words.^15^ These are analyzed with OLS linear regression with predictor *GTIdest*_*jat*_. Furthermore, the statistical association between the Google Trends results of the third category (*jobs and education*) and educational-specific migration data by JASSO for the U.K. is analyzed.

Next, the first analysis is repeated but the country names are exchanged with a key city from each country. As reflected in the literature, cities such as Paris, Düsseldorf or Brussels are known within their respective countries and Japan as featuring a relatively established Japanese community and may serve as a prime destination for Japanese immigrants. We examine if these city names can serve as proxies for country names. Some keywords practically make more sense on a regional/city level. For instance, when searching for accommodation it can be assumed that people do this at the level of a city and do not just look for a place to stay anywhere in the country. Here we focus on one predictor *GTIbil*_*jat*_ and analyze the statistical association via OLS linear regression for 2011–2019.

Finally, as a fifth step, the search location is changed from Japan to each of the five European countries (*cod*). This translates into searching how frequently Japanese words were searched for in European countries. In this step, only the Japanese keywords are used without the European country or city name. These are analyzed for both periods starting in 2006 and 2011 via OLS linear regression with predictor *GTIuni*_*codt*_. The inspiration for this reversed approach can be found in Connor’s research (Connor 2017). We assume that after people have moved, they still need to search for information that may be captured by Google (e.g., where the embassy is to arrange visa formalities, looking for a job, how tax works, and more).

Throughout the above analyses, linear regression is used for a number of reasons. One of which is that linear regression is used in comparable research primarily focusing on Google Trends as an alternative data source (Böhme et al. 2020; Wanner 2021). In addition, with the exception of Belgium, the lack of accurate monthly migration flow data prevented more nuanced analyses (such as time series) in which potential within-year seasonality could be uncovered and examined. Furthermore, this research is primarily concerned with the suitability of Google Trends in a different, as of yet unexamined context rather than pursuing innovation in how this data can be used in advanced statistical modeling.

Another reason is that it conceptually follows the logic of migration aspirations: More people aspiring to migrate means more people searching for information. Increases/decreases in these numbers ought to be followed by increases/decreases in real mobility, potentially after some delay. Whereas other research makes use of a narrower, more targeted range of methods and data, there is no prior research that can be used as a guideline for analyzing Japanese immigration. Consequently, this research opts to explore several ways of searching for statistical association by using a wide range of Google search terms.

## Results

### Google Trends and the Japanese writing system

#### 5.1.1. Kango or Sino-Japanese vocabulary

Google searches of Sino-Japanese words (24 out of 30) are predominantly performed in *kanji* (see Table 2). For 13 out of 24 keywords, the search frequency in Google for inputs in *hiragana*, *katakana*, and *rōmaji* is equal to or lower than 1 on a scale from 0 to 100 which means they are barely used relative to the *kanji* version. Seven of the 24 keywords are predominantly searched for in *kanji*, but also show some frequencies for inputs in *hiragana* albeit much lower. Each of the remaining four variations is unique in the sample of keywords: one keyword is not searched for enough so there is no result in Google Trends, another features some small fluctuations not only in *hiragana* but also *katakana*, and a third also in *rōmaji.* A final keyword, *kika*, meaning ‘naturalization’ (of, for instance, citizenship), results in more *hiragana* than *kanji* searches. From this initial analysis, we conclude that for *kango* or Sino-Japanese word searches in Google Trends the predominant writing system for Japanese input is *kanji*.

**Table 2:** Number of successful Google Trends keyword extractions per writing system

1. Only *kanji*	13
2. Predominantly *kanji*, and small fluctuations in *hiragana*	7
3. Predominantly *kanji*, and small fluctuations in *hiragana*, *katakana*, and *romaji*	1
4. Predominantly *kanji*, and fluctuations in *hiragana* and *katakana*	1
5. Predominantly *hiragana*, and fluctuations in *kanji*	1
6. No result	1
Total	24

#### Katakana loanwords

The list of words for this category is more limited and includes loanwords such as ‘visa’, ‘hotel’, or ‘internship’. Here, transcription in *kanji* is not possible (a *kanji* equivalent does not exist), so only a comparison with *hiragana* and *rōmaji* can be made. Google searches of these kinds of words appear to be done overwhelmingly in *katakana*. Both inputs in *hiragana* and *rōmaji* do not show up in Google Trends.

For the next steps of the analysis, Sino-Japanese words can be input in *kanji*, and loanwords in *katakana*.

### Predicting migration with Google Trends data

In this section, first the results of the search actions of keywords in Google Trends are discussed, followed by the potential for predicting migration with these data.

#### Searching for migration

The number of positive hits, that is when the input of the keyword (and country/city) in Google Trends generates a usable time series of relative search frequencies, depends on the country or city name used. Bilateral searches (both place name and keyword combined—*GTIbil*_*jat*_) have the highest success rates (56%) for France and Germany. Belgium, although similar to the Netherlands in terms of population size, has a much lower success rate than its Northern neighbor in generating usable Google Trends data (10% vs 29%). Combinations with city names instead of country names, have a low success rate for Amsterdam, Brussels, and Düsseldorf (2% to 4%), whereas Paris and London score relatively well (33% and 36% respectively). Focusing only on the country name (*GTIdest*_*jat*_) or the keywords (*GTIuni*_*jat*_) always generated results. Finally, when searching for just the Japanese keywords but changing the search location from Japan to the European countries of destination (*GTIuni*_*codt*_), there was a low success rate for Belgium (9%) in generating usable time series, and higher rates for the other four countries, with the United Kingdom on top (67%) (see supplementary data).

#### Predicting migration

**Fig. 2:**
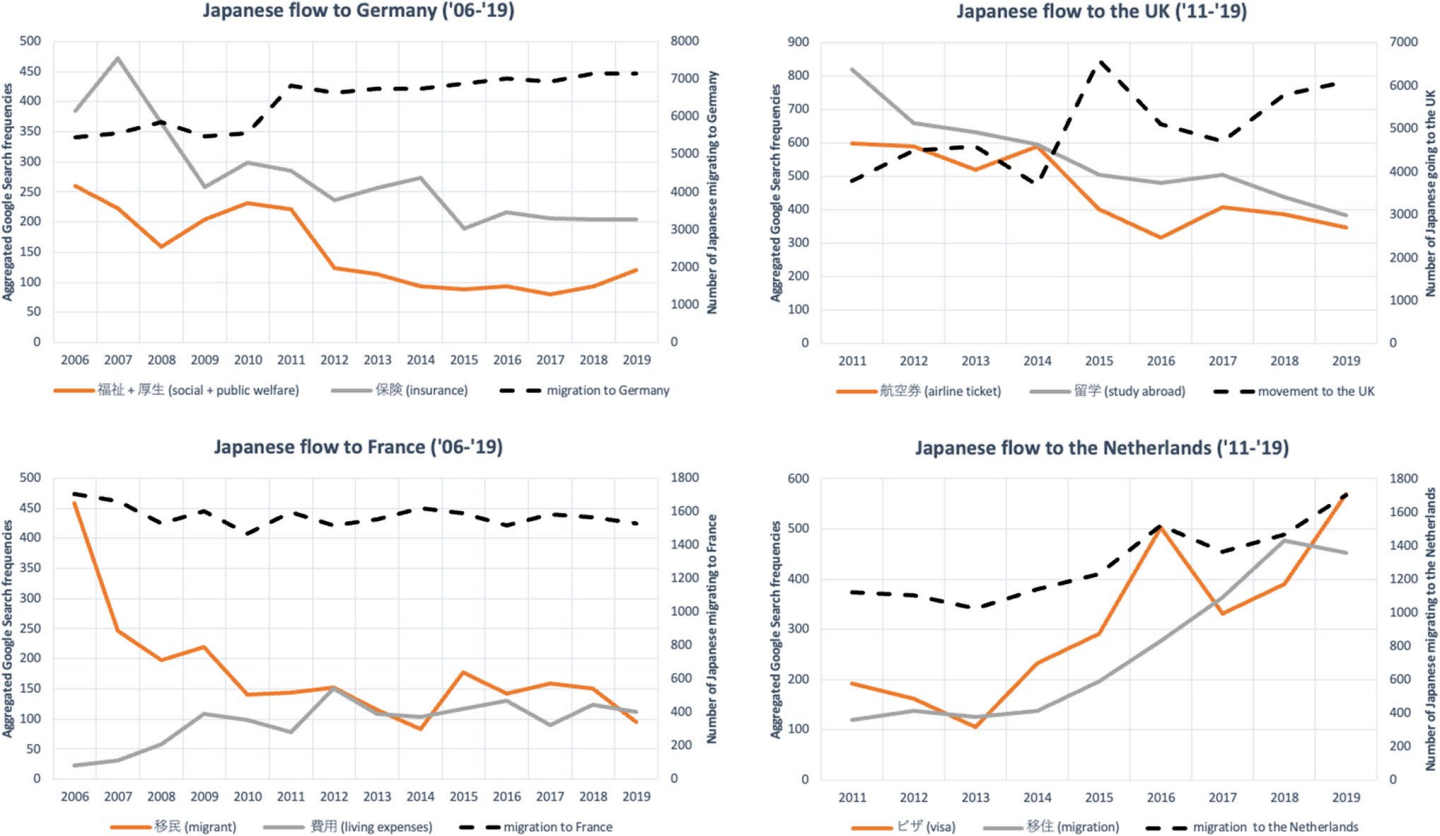
Japanese migration flows to Germany, France, the UK, and the Netherlands (black dotted lines) versus top keywords

Following the mixed quality of data that are extracted from Google Trends, we can expect considerable differences between the countries in coefficients of determination (R^2^ values), indicating correlations between searches (and aspirations) and mobility. In Table 3 only keywords with the highest R^2^ are included to maintain the overview. First, the correlations between the keywords and country names, and Japanese immigration flow figures are examined (analysis I). Overall, the associations between searches and Japanese migration flows are best for Germany and the Netherlands. For Germany, the highest R^2^ value of 0.678 is for the search terms related to ‘welfare’ from 2006 through 2019, 0.697 is for ‘visa’, and 0.621 for a compound keyword consisting of ‘applicant + recruitment + employment’ in the period 2011–2019. For the Netherlands in the period 2006–2019, we note the highest R^2^ for the search term capturing various configurations of the term ‘migration’ (0.686). The results for the period 2011–2019 show an R^2^ of 0.969 for ‘visa’ and 0.787 for ‘migration’ (see Fig. 2 for a visual comparison of the relative search frequencies of several keywords with comparatively high R^2^ values, and migration flows).

**Table 3:** results of the OLS models for keywords combined with country names in period (predictor GTIbiljat; dependent variable: number of moves from Japan to European countries)

Search period	Location keyword	Keyword	Constant	Regression coefficient 1	R^2^
2006–2019	France	移民 (migration)	1488.650***	0.507**	0.522
	Germany	福祉 + 厚生 (social welfare + public welfare)	7734.545***	− 8.684***	0.678
		保険 (insurance)	8192.462***	− 6.415**	0.597
	The Netherlands	移住 (migration)	989.509***	1.682***	0.686
2011–2019	Germany	ビザ (visa)	6350.124***	1.361**	0.697
		応募者 + 募集 + 採用 (applicant + recruitment + employment)	7693.474***	− 2.617*	0.621
	The Netherlands	ビザ (visa)	850.448***	1.444***	0.969
		移住 (migration)	938.179***	1.410**	0.787
	United Kingdom	航空券 (airline ticket)	8185.318***	− 6.907*	0.598
		留学 (studying abroad)	8156.711***	− 5.671*	0.567

Significance: ***p < 0.001; **p < 0.01; *p < 0.05

More surprising is the lack of correlations found for keywords combined with ‘France’. Despite the high success rate in extracting data from Google Trends, only the Japanese word ‘migration’ resulted in an R^2^ higher than 0.5 (0.522). Lastly, when examining the correlations of Google searches and migration flows to the United Kingdom, only ‘airline ticket’ and ‘studying abroad’ resulted in R^2^ values higher than 0.5 for the period 2011–2019 (0.598 and 0.567 respectively). Specifically for the analysis of educational mobility to the U.K., the highest R^2^ values were recorded for the keywords ‘studying abroad’ (0.739) and a combination of two words for ‘airline ticket’ (0.854).

While R^2^ values are informative and have been used in prior research to indicate correlations between Google Trends and migration, they do not explain the complete situation. For the logic of migration aspirations which are translated into search action and movement to make sense in this analysis, the regression coefficient should be positive since more searches lead to more movement. Table 3 shows this is not always the case. Negative coefficients are interspersed with positive ones, signifying that sometimes a *higher* search frequency correlates with a *lower* movement. Negative coefficients are also present when examining educational mobility specifically. Although adjusted coefficients of determination show a statistical association, the negative coefficients illustrate an inverse nature and thus contradict the search to movement logic.

In the second approach, a predictor in the form of the interaction term *GTIuni*_*jat*_ x *GTIdest*_*jat*_ is added and the correlations with official immigration figures are examined. Table 4 shows the overall fit in most cases improving by adding this interaction term. For Germany, there are four words with a coefficient of determination above 0.8 in the 2006 period. And whereas for the 2011 dataset the prediction power is lower, there are significantly more words that have a high R^2^ value compared to just the predictor *GTIbil*_*jat*_. The statistical association for the Netherlands is higher as well, but for several words, the added interaction term does not increase prediction power (e.g., ‘visa’ or ‘migration’). The results for France and the United Kingdom are also mixed. The regression coefficient for the first predictor again shows opposite signs for some words, and the second predictor mainly shows coefficients of zero or close to zero. Therefore, these belie the fact that all keywords capture the same aspirations.

**Table 4:** results of the OLS models for keywords combined with country names in period 2006/2011–2019 (predictors GTIbiljat and GTIunijat × GTIdestjat; dependent variable: number of moves from Japan to European countries)

Search period	Location keyword	keyword	Constant	Regression coefficient 1	Regression coefficient 2	R^2^
2006–2019	France	航空券 (airline ticket)	1518.312***	− 0.644**	0.000***	0.705
		大使館 (embassy)	1538.772***	− 0.576*	0.000**	0.556
	Germany	求人 (recruiting)	4162.527***	0.418	0.006***	0.886
		求人 + 欠員 + 空席 (recruiting + vacancy)	4298.163***	− 0.194	0.006***	0.879
		航空券 (airline ticket)	7463.441***	5.933**	− 0.006***	0.865
		仕事 (job, work)	3945.387***	2.048	0.005**	0.806
	The Netherlands	採用 (employment, recruitment)	1830.502***	− 2.057*	− 0.003**	0.693
		移住 (migration)	956.093**	1.661**	0.000	0.686
	United Kingdom	航空券 (airline ticket)	5990.619***	− 18.721**	0.010	0.576
2011–2019	Germany	領事館 + 大使館 (consulate + embassy)	7261.092***	2.052*	− 0.006**	0.796
		ビザ + 査証 (visa + visa)	7120.994***	1.726**	− 0.002*	0.794
	The Netherlands	ビザ (visa)	870.626***	1.435***	0.000	0.969
		航空券 (airline ticket)	2347.759***	− 1.391	− 0.003*	0.801
	United Kingdom	無職 + 失業 (unemployment + unemployment)	− 358.186	− 8.170*	0.020	0.612

Significance: ***p < 0.001; **p < 0.01; *p < 0.05

When repeating this analysis after introducing lag between searching and moving (Table 5), there is an increase in the coefficient of determination for Germany, reaching the levels of prediction found by Golenvaux et al. (2020) and Böhme et al. (2020) (e.g., for ‘economy’). However, the coefficients are predominantly negative. So, translating this to searching and migrating would mean that *more* searches of these keywords result in *less* migration. For France, prediction power of the keywords decreases when the period 2006–2019 is analyzed but increases for the period 2011–2019 with the words for ‘moving (between houses)’ and ‘contract’ resulting in an R^2^ value of 0.838 and 0.826 respectively. For the Netherlands, some words switch places in the ranking: ‘visa’ was the best predictor in the previous models but now ranks last among those words with a coefficient of determination higher than 0.5. ‘Employment’ now has the best statistical association (0.832). Adding lag for the United Kingdom has mixed results. The overall statistical association remains mediocre, but the successful words are more work-related.

**Table 5:** results of the OLS models with 1 year lag for keywords combined with country names in period 2006/2011–2019 (predictors GTIbiljat and GTIunijat × GTIdestjat; dependent variable: number of moves from Japan to European countries)

Search period	Location keyword	Keyword	Constant	Regression coefficient 1	Regression coefficient 2	R^2^
2006–2019	Germany	募集 (recruitment)	9438.609***	− 5.645**	− 0.005***	0.863
		領事館 (consulate)	7851.748***	− 3.459	− 0.003^*^	0.796
		経済 (economy)	8184.233***	− 0.844	− 0.004**	0.782
		航空券 (airline ticket)	7589.779***	4.653*	− 0.006***	0.777
	The Netherlands	移住 (migration)	895.214**	1.798**	0.001	0.764
2011–2019	France	移動 (moving)	1891.162***	0.424*	− 0.001**	0.838
		契約 (contract)	1951.272***	0.051	− 0.001**	0.826
		勉強 (study)	1574.754***	0.491	0.000**	0.736
	Germany	経済 (economy)	8047.301***	− 1.780*	− 0.001	0.919
		インフレ (inflation)	7653.390***	− 0.911	− 0.002	0.846
		採用 (employment)	7631.674***	0.381	− 0.003**	0.843
	The Netherlands	採用 (employment)	2052.931***	− 3.111*	− 0.002	0.832
		移住 (migration)	890.031*	1.469*	0.001	0.754
	United Kingdom	福祉 + 厚生 (social welfare + public welfare)	10,732.958**	− 25.379*	0.005	0.771
		求人 + 欠員 + 空席 (recruiting + vacancy)	6583.930*	− 9.977*	0.007	0.630

Significance: ***p < 0.001; **p < 0.01; *p < 0.05

In a third analysis, we examine if migration can be predicted by relying on Google’s in-built algorithm instead of combining place names with keywords. For this, search data in specific categories as designated by Google are extracted (see supplementary data). While we managed to extract time series from Google Trends for each instance, none of the coefficients of determination are high. Only searches for ‘Germany’ for the period 2006–2019 in the categories jobs and education and law and government result in an R^2^ value higher than 0.5 (0.582 and 0.541). However, in this case, the regression coefficients are negative (-5.741 and -7.648), thus not matching the hypothesis that more Google searches, as a proxy for migration aspirations, result in more mobility. More successful seems the pairing with student mobility data to the U.K. The category of *jobs and education* resulted in a statistical association with an R^2^ of 0.903. However, here too, the regression coefficient is negative.

Following the fourth approach, the first analysis is repeated but country names are replaced by city names Amsterdam, Brussels, Düsseldorf, London, and Paris (see supplementary data). Compared to the countries (except for Belgium), cities show a lower statistical association. Only for London can we find a correlation with an R^2^ value above 0.5 (0.545 for the keyword ‘studying abroad’). For the other search terms, the quality of the data was too low to conduct any meaningful analysis (i.e., containing a large number of months with 0 relative search frequencies).

The last analysis substitutes Japan as the search location in Google Trends for the European countries of destination (Table 6). The results regarding the strength of prediction are in line with those of the first analysis. Only several keywords result in fairly high R^2^ values. Perhaps more strikingly, the regression coefficients are all positive and consequently fit the search-to-action hypothesis. The best results are for Germany and the Netherlands for the words ‘money’ and ‘moving’ (R^2^ of 0.769 and 0.750) and ‘work’ or ‘job’ respectively (R^2^ of 0.577 and 0.808 for the periods starting in 2006 and 2011 respectively. It should be noted that most of the words that result in statistical association differ from those in the earlier analyses. The association for the United Kingdom and France is likewise poor, with R^2^ values staying below 0.6.

**Table 6:** Results of the OLS models for different keywords searched for in the European country of destination (predictor GTIunicodt; dependent variable: number of moves from Japan to European countries)

Search location	Search period	Keyword	Constant	Regression coefficient	R^2^
Germany	2006–2019	お金 (money)	5536.400***	4.341**	0.594
		日本人 (Japanese person)	4039.580***	4.523**	0.587
		差別 (discrimination)	5622.890***	3.794**	0.516
		移動 (moving)	5114.533***	3.842**	0.506
		ビザ (visa)	4904.433***	4.540**	0.505
	2011–2019	お金 (money)	6566.163***	1.017**	0.769
		引っ越し (moving)	6625.974***	1.436**	0.750
		入国 (entry into a country)	6452.448***	1.466**	0.717
		費用 (living expenses)	6561.573***	0.913**	0.656
		切符 + チケット (ticket + ticket)	6529.261***	0.917**	0.646
		差別 (discrimination)	6586.201***	1.015*	0.542
The Netherlands	2006–2019	ビザ (visa)	896.938***	3.306**	0.645
		仕事 (job, work)	1016.628***	1.858**	0.577
		勉強 (study)	1014.923***	1.877**	0.506
	2011–2019	仕事 (job, work)	1048.083***	1.128**	0.808
		費用 (living expenses)	1052.306***	1.178**	0.691
		採用 (employment)	1093.066***	1.189*	0.512
United Kingdom	2011–2019	無職 + 失業 (unemployed + unemployed)	3652.558***	6.171*	0.594
France	2011–2019	日本人会 (Japanese expat organization)	1468.795***	0.303*	0.512

Significance: ***p < 0.001; **p < 0.01; *p < 0.05

In none of the above analyses was there any useable result for Belgium which can be expected considering the low number of useable keywords found before.

## Discussion and conclusion

This study aims to examine Japanese immigration to Europe as a new context in which to empirically investigate alternative data sources such as Google Trends. A first aim was to examine to what extent language writing systems impact online searches conducted with Google, and by extension the data used for estimating immigration flows. Next, we examined whether Google Trends data can function as a tool for predicting Japanese immigration flows to European countries signaling migration aspirations.

The findings indicate that the specific writing system is of consequence in this case. For non-Latin scripts, it is advisable to conduct a preparatory study of the different writing systems, especially if the researcher is less familiar with its peculiarities. While the results for Japanese suggest that the writing system we logically expect for each word can be safely used (e.g., *kanji* for Sino-Japanese words, and *katakana* for loanwords), there are exceptions. For instance, the word きか (*kika*) featured a higher frequency in *hiragana* than the *kanji* equivalent 帰化 which can be explained by the significant number of homophones for this word. In other words, it is plausible that people searching in Japanese use the *hiragana* form because the correct *kanji* are less known.

In all, research that uses Google Trends data would benefit from the additional step of analyzing the different ways of inputting keywords. This conclusion is a tentative one and may be specific to the Japanese language. Google’s algorithms are proprietary, so it is difficult to come to generalizable findings. Also, this challenge can lessen over time as AI-powered translation engines become more powerful and input in different writing systems may become combined in singular search terms.

Compared to similar exercises in other research (Böhme et al. 2020; Golenvaux 2020; Wanner 2021) the success rate of the prediction analyses here is lower, including the specific analyses focused on student populations migrating to the U.K. A first contextual limitation can be found in the immigration flows from Japan. Not only are these flows rather stable with little variation, but they are also limited compared to the total Japanese population (126 million in 2019, World Bank). This first point makes statistical analysis challenging. The second point is relevant for using Google Trends since it relies on relative search frequencies. As such, the keywords need to be searched sufficiently compared to all other Google searches within Japan to generate usable results. Consequently, if a thousand people (roughly the yearly flow from Japan to the Netherlands or Belgium) Google information related to migrating, this could be too small a number compared to 126 million people Googling other things. This explains why several Google Trends keyword extractions were not successful.

There is also a cultural aspect to a certain subsection of Japanese migration which may limit the usability of this investigation. Japanese companies are highly regulated, compartmentalized, and process-bound (Fulcher 1988; Shimizu 2020). Consequently, most aspects of company and even private life are taken care of by departments rather than the individual. Specifically for expats, it is primarily the HR department that is in charge of the preparations for employees moving to Europe. These departments in turn consult specialized firms that deal with the paperwork for visa applications, shipping personal items and more. The specialists employed by these firms may have little need for Google: Contacts at the embassy may be stored in Outlook, so there is no need to Google ‘embassy’ to look up contact information. Draft and blank forms for visa applications and templates for moving companies may be stored on a local server, similarly bypassing the need for Google search. This may explain the low search frequencies and consequently the lack of predictive power of Google Trends for Japanese immigration flows. However, these limitations are not yet substantiated by any further empirical investigation so they should be approached with care.

Furthermore, the relevance of certain keywords to migration can be put into question. Words such as ‘customs’ may not only capture the search results of someone intending to migrate, but also someone trying to find out if there is an additional tax on a shipment from the UK, France, or any other country. Similarly, words such as ‘airline tickets’ or ‘hotel’ capture the search actions of migrants as well as those of tourists potentially creating significant bias. When possible, it is recommended to use words that can only (or predominantly) be linked to migration. Japanese tourists do not need a visa to visit the countries analyzed here. So we can be fairly certain to have captured long-term sojourners rather than tourists with these keywords.

With regards to methodological limitations, the analyses performed here are relatively straightforward. More complex modeling may reveal other useful aspects such as reductions in estimation errors which are more difficult to identify here (for instance, see Böhme et al. (2020) and Golenvaux et al. (2020)). In addition, a key limitation is that the OLS models used fail to capture within-year seasonality or specific lags (for instance, Japanese expats may disproportionately arrive by the end of the year when they are assigned new positions). It is highly recommended to control for these aspects in case accurate monthly immigration data can be obtained. Future research may also opt to focus on countries with available monthly data to examine the different lags more in detail and obtain an overall more thorough picture of migration flows.

Aside from these limitations, we identify several findings. First of all, in the analyses, the useful keywords differ between countries. The word ‘visa’ is a strong performer, resulting in the highest predictive value for the Netherlands (0.969) but less so for other countries. This shows the need to carefully curate keywords, and not to rely solely on lists generated by previous research or online tools.

Also, this method can work for smaller migration flows. The strongest predictor was found for the Netherlands despite its much smaller immigration flow from Japan (on average yearly 1296 people compared to 6898 to Germany over the same 15-year period. While it was more challenging to find usable Google search data for smaller countries, carefully curating keywords can result in good statistical association.

In addition, ‘visa’ resulted in a good prediction for the period 2011–2019 for the Netherlands but was not usable for the period 2006–2019. Similarly, when the interaction term was added, we found fewer results for the United Kingdom during the period starting in 2006 compared to the period starting in 2011. This shows that the time period used to analyze Google Trends data can have a distinct impact on predictive power, and consequently on research using these data in general.

Lag between search and movement also had significant effects. For France (2011–2019), the predictive power increased substantially after introducing a 1-year lag. We also see that the most suitable words change after introducing this lag. In the Netherlands, words such as ‘employment’, ‘economy’, and ‘expenses’ seem to matter more *before* mobility, whereas without lag words such as ‘visa’ and ‘airline ticket’ suit the immigration data better. This finding may substantiate the hypothesis that specific keywords reflect specific phases leading up to migration (changing aspirations). Words such as ‘employment’, ‘economy’, and ‘expenses’ signify a preparatory phase with individuals researching the future location (how is the economy, the employment situation, etc.) thus signaling immigration aspirations. Words such as ‘visa’ and ‘airline ticket’ searched later may signal increasingly crystalizing aspirations, leading to concrete plans and preparations (e.g., getting a visa, buying a plane ticket). With careful tuning, it appears Google Trends data hold promise as a data source contributing to the expansion of the aspirations-(cap)ability approach as proposed by Carling (2002) and de Haas (2014; 2021).

Analyzing Japanese words in the countries of destination was on average more successful, particularly considering the overall positive regression coefficient. This finding links back to the first research aim concerning language. Not only does the input matter, but also where we search. It is possible to predict migration by using a language or writing system not used by the local population to identify migrant groups and their search actions, echoing some of Connor’s findings (Connor 2017). A possible explanation for this may be that some information is (also) searched after arrival (e.g., opening hours of the embassy or job positions). Part of this could also be explained by expats searching for information for family members who often join them with some delay. Focusing on this type of analysis where keywords in the language of the migrant are examined in the arrival country is a promising avenue for future research.

Using main cities of destination as proxies for country names or just using country names and Google Trends categories to capture related search queries produced no usable results. This may be due to the relatively small immigration flows and could work better for forecasting tourism and shorter stays in Europe (Li et al. 2021). Also, despite having a similar Japanese immigration flow to the Netherlands, Google search frequencies and predictions for immigration to Belgium were poor in all five analyses.

Perhaps the strongest admonition is that the effect of search frequencies on mobility was often not positive. For some cases, we can imagine how increased searches may capture (or result into) anxiety about mobility. Information may confront a searcher with potential difficulties. For instance, job hunting in Japan differs considerably from that in Europe. Searching for specific procedures online may put potential applicants off, resulting in a repression of migration aspirations. Furthermore, most of these negative effects were found in the interaction term (*GTIuni*_*jat*_ × *GTIdest*_*jat*_) which does not specifically capture the keywords in conjunction with the migration location as the predictor (*GTIbil*_*jat*_) in the first analysis does. Considering the small migration flows, a completely unrelated trend may be captured by this predictor.

Lastly, a broader limitation regarding the proposed method needs to be mentioned as well. Although the current market share of Google as a search engine is currently high for Japan, there is no guarantee this will remain the case or is the case in all countries.^16^ Countries such as China and Russia have their own search engines (e.g. Baidu, Petal Search, or Soguo in the former and Yandex in the latter), so only using Google data will not be representative or even possible in these cases. In addition, in countries where Google is the main search engine, it also faces competition. Especially against the background of increasing awareness and concern among the public regarding privacy (in particular aimed at large tech companies like Facebook or Google), it remains to be seen if this method stays valid for future research. Certain tracking functions can be switched off more easily now, and the use of VPNs is also becoming more widespread. The emergence of privacy-conscious search engine alternatives, such as DuckDuckGo, or increasingly easy-to-access and use privacy-centered web browsers (e.g. Tor, Brave, or Firefox Focus) may pose an additional challenge for future research. A low market share would increase potential selection bias significantly and can render the method useless.

Perhaps the highest risk to future research is not posed by Google’s potentially decreasing market share and consequently the representativeness of the data, but by the fact that Google remains a profit-oriented company. To offer the service of Google Trends (for free) is consequently subject to its business prospects and can be canceled at any time without prior warning. Especially for projects and research that requires continuous up-to-date data, this can be a significant limitation to using Google Trends data as a reliable alternative data source.

In conclusion, Google search data can be used as an alternative data source for predicting migration, but there are challenges depending on the context. The current research presents a specific case with mixed results. While we find it is possible to predict some migration flows from Japan to Europe, this prediction power is highly specific to destination, time frame, and in particular, the keywords used. Certain cultural limitations which are absent in previous research should be carefully considered as well, thus making migration research with Google Trends a worthwhile but challenging option.

## Supplementary Information

Below is the link to the electronic supplementary material.

Supplementary file1 (DOCX 95 kb)

## Footnotes

1 With the exception of names and romanization methods preferred by original sources, this paper uses the Traditional Hepburn romanization of the Japanese language. All translations are done by the author.

2 This is an important point of access for developing regions which increasingly rely on mobile internet infrastructures.

3 It should be noted that Google’s market share at the time of their investigation only reached approximately 45%, being outperformed by Russia’s domestic provider Yandex. This naturally has implications on the external validity of the data.

4 The United Kingdom’s Office for National Statistics has monthly numbers, but these are not split between citizenship or countries of origin and are consequently not suitable for this paper.

5 An exception are the numbers for asylum seekers and refugees which are monitored more closely.

6 The immigration flow from Japan to Germany for 2019 was lacking in the OECD dataset at the time of consultation and was supplemented with data generated by the German Federal Statistical Office (set 12,711-0007).

7 https://gs.statcounter.com/search-engine-market-share/all/japan retrieved on January 8, 2022.

8 There is a small dip around 2015 when Yahoo! gained market share (Google down to 60%, and Yahoo! up to 37%). However, this trend was reversed quickly and in the past two years Yahoo! represents only 18% (https://gs.statcounter.com/search-engine-market-share/all/japan#monthly-200901-202208 retrieved August 18) Statista shows a similar situation with regards to market share (https://statista.com/statistics/1173906/japan-leading-internet-search-engines retrieved on August 12, 2022).

9 https://statista.com/statistics/1173906/japan-leading-internet-search-engines/ retrieved on August 18, 2022).

10 Some selection bias is expected due to differences in digital literacy and access to technology. This second part, however, is not a concern when dealing with Japan. According to the ITU (International Telecommunication Union), a specialized department for information and communication technologies by the UN, Japan has a 3G mobile coverage of 100%, and a 4G mobile coverage of 99% of the population (2019 and 2017 respectively), so the basic network is well established. Active subscriptions follow the same trend: 203 active mobile-broadband subscriptions per 100 inhabitants and 34 fixed subscriptions per 100 inhabitants in 2019 (International Telecommunication Union 2021).

11 These are words with a different meaning but the same or similar sound.

12 Whereas the basic sets (roughly 2000 characters) are learned in school, a complete list of *kanji* existing in Japanese would range from 40,000 to more than 75,000 unique characters. Diverging proficiency is illustrated by a nationally organized kanji-exam (*kanji kentei*) aimed at Japanese of all ages and levels. 631,521 people registered for the second round in 2020, but only 10.9% could pass the most difficult first grade (Nippon kanji nōryoku kentei 2020 nendo (2020).

13 For Belgium, the monthly time series are used. For other countries these are aggregated to yearly ones.

14 We only follow the researchers’ principle of constructing Google Trends indexes but not the analysis since they used Google Trends as part of a model rather than by itself. In this research, we are more concerned with the keywords, so our emphasis differs.

15 We explored the variation of these categories also with the set of key terms but found that Google would restrict the searches to a level that most results became unusable. As such, we do not cover this side-investigation here.

16 Depending on the source, the market share for Google in Japan fluctuates between ca. 70% (Statcounter) and 76% (Statista) in the period 2009–2022.
