# Supplementary material for: Searching for migration: estimating Japanese migration to Europe with Google Trends data
Source: Qual Quant. 2022 Nov 14:1–29. Online ahead of print. doi: 10.1007/s11135-022-01560-0 (PMC9662772; doi:10.1007/s11135-022-01560-0)
Supplement: Supplementary file 1 — Supplementary file1 (DOCX 95 kb) [file 11135_2022_1560_MOESM1_ESM.docx]

**SUPPLEMENTARY INFORMATION**

Homophones for the word pronounced as kōkan

The list with words in this table displays homophones for what would be pronounced as *kōkan*. In *hiragana* the different meanings are unclear, whereas in *kanji* they are.

Success rates for extracting Google Trends data for different country names, city names, and in different countries

Source: compiled based on data extracted from trends.google.com
The absolute number of words are based on a set of 90 words times two to simulate the different time periods analyzed.

Results of the OLS models with the European country name as keyword searched for in Japan as categorized by Google (dependent variable: number of moves from Japan to European countries)

Results of the OLS models for the different keywords combined with different city names (predictor GTIbil_jat_; dependent variable: number of moves from Japan to European countries)
